# Integration of bilateral nociceptive inputs tunes spinal and cerebral responses

**DOI:** 10.1038/s41598-019-43567-y

**Published:** 2019-05-09

**Authors:** Nabi Rustamov, Stéphane Northon, Jessica Tessier, Hugues Leblond, Mathieu Piché

**Affiliations:** 1 Department of Anatomy, Université du Québec à Trois-Rivières, 3351 Boul. Des Forges, C.P. 500, Trois-Rivières, QC G9A 5H7 Canada; 2 CogNAC Research Group, Université du Québec à Trois-Rivières, 3351 Boul. Des Forges, C.P. 500, Trois-Rivières, QC G9A 5H7 Canada

**Keywords:** Reflexes, Reflexes, Sensory processing, Sensory processing, Neurophysiology

## Abstract

Together with the nociceptive system, pain protects the body from tissue damage. For instance, when the RIII-reflex is evoked by sural nerve stimulation, nociceptive inputs activate flexor muscles and inhibit extensor muscles of the affected lower limb while producing the opposite effects on the contralateral muscles. But how do the spinal cord and brain integrate concurrent sensorimotor information originating from both limbs? This is critical for evoking coordinated responses to nociceptive stimuli, but has been overlooked. Here we show that the spinal cord integrates spinal inhibitory and descending facilitatory inputs during concurrent bilateral foot stimulation, resulting in facilitation of the RIII-reflex and bilateral flexion. In these conditions, high-gamma oscillation power was also increased in the dorsolateral prefrontal, anterior cingulate and sensorimotor cortex, in accordance with the involvement of these regions in cognitive, motor and pain regulation. We propose that the brain and spinal cord can fine-tune nociceptive and pain responses when nociceptive inputs arise from both lower limbs concurrently, in order to allow adaptable behavioural responses.

## Introduction

Nociception and pain constitute an alarm system that detect actual or potential damage to body tissues, which evokes well characterized spinal^1,2^ and cerebral^3–6^ responses. When nociceptive inputs arise from multiple sources, however, information must be integrated by the central nervous system to produce adapted spinal reflexes and coordinated behavioural responses. This is critical for body integrity and survival, but the underlying mechanisms of this integration has never been investigated and remains unknown.

Spinal reflexes evoked by limb stimulation include a variety of responses to non-nociceptive and nociceptive stimuli. They have beed investigated extensively in animals since Sherrington^7–9^, who first described the flexor reflex afferent (FRA) system responsible for withdrawal reflexes that protect limbs from nociceptive stimuli. In spite of some exceptions, he showed that nociceptive inputs activate flexor muscles and inhibit extensor muscles of a lower limb (flexion reflex) while producing the opposite effects on the contralateral flexor and extensor muscles (crossed-extension reflex)^8,9^. Later, Lundberg and his group defined the FRAs as afferents that may evoke the flexion reflex^10^ and which associated interneurons produce spinal reflexes that are regulated by supraspinal structures^11^. Importantly, the FRA system was broadened to include afferents and pathways that are not specific to nociception, in which interneurons receive multisensory convergence for the control of movement^12–14^ (also see review by^15,16^). In the present study, we studied a pathway specific to the RIII-reflex and not the broader, “nonspecific” FRAs system described by Lundberg’s group^11–14^ and discussed in Rossi^17^, which includes several pathways (for an extensive review see^15^). Nociceptive inputs producing the RIII-reflex may certainly converge on FRA interneurons, but are not equivalent to what is referred to as FRA reflexes, which are produced by a variety of inputs, nociceptive or not. Accordingly, as shown by Steffens & Schomburg^18^, nociceptive afferents are constituents of the FRAs that add a nocifensive function to the FRA system, the later being dedicated to the control of movement and not specifically to withdrawal and protection.

In the animal, each of the hindlimb muscles has a highly organized cutaneous receptive field, which causes the withdrawal of the stimulated receptive field independently of the position of the limb^19^. The final withdrawal movement is usually due to activation of several muscles resulting from overlapping receptive fields. Thus, convergence of a large number of afferent fibres onto the reflex pathways occurs on spinal interneurons^20–22^. In human, reflexes produced by the FRA system are considered as responses distinct from cutaneomuscular reflexes and the RIII-reflex^16^, the later being referred to as a nociceptive flexion reflex^1^. The RIII-reflex was investigated by Hagbarth^23^ and Hugon^24^ and it was shown that nociceptive stimuli usually evoke ispilateral flexion, but extension may also occur if the skin overlying flexor muscles is stimulated. This is why the more general term “withdrawal reflex” is sometimes used to refer to responses evoked by nociceptive stimulation of the limbs. Nevertheless, the RIII-reflex produced by stimulation of the skin overlying the sural nerve in its retromalleolar pathway always consists in activation of the biceps femoris and flexion of the knee^1^. In addition, this response relies on A-delta fibers and is strongly associated with pain perception^1,25,26^. Using similar methods, it was shown that ipsilateral flexion may be associated with contralateral extension^27^. These responses allow withdrawal of the limb affected by nociceptive stimuli and weight bearing by the contralateral limb. Thus, concurrent nociceptive stimulation of both lower limbs is expected to decrease RIII-reflex amplitude. In contrast to the inhibitory interaction of contralateral inputs, neurophysiological studies indicate that some peripheral afferent fibers have excitatory projections to the contralateral spinal cord^28^. These functional observations are supported by anatomical studies showing projections from peripheral afferents to the contralateral spinal cord^29^. Together, these findings suggest that spinal nociceptive reflexes may be modulated by competing inhibitory and facilitatory inputs from ipsilateral and contralateral afferents, in addition to modulatory influences from supraspinal pathways. However, how the spinal cord integrates competing nociceptive inputs remains to be clarified and has never been investigated in human. This may reveal novel pain mechanisms that are essential to elicit adapted nociceptive reflexes and behavioural responses, depending on the sources of nociception.

Brief painful stimuli elicit cerebral activity measured as event-related potentials (ERP). The amplitude of ERP elicited by nociceptive stimuli is known to be affected by several factors, including stimulus saliency and spatial attention. For instance, when a stimulus becomes less salient, ERP amplitude is decreased^30^. This may occur with concurrent painful stimuli since each competing stimulus becomes relatively less salient. This would also be consistent with the modulation of ERP amplitude by spatial attention^31^. However, it is also possible that two concurrent painful stimuli may increase saliency if perceptual fusion occurs^32^, resulting in a unique coherent percept of increased threatfullness. In this case, ERP amplitude is expected to increase. To date, however, the integration of inputs from concurrent painful stimuli on different body parts and how this is reflected in ERP amplitude (decrease or increase) has never been investigated. This integration may be an essential neural process in response to pain perception in order to produce the most adapted behavioural responses.

Painful stimuli also elicit event-related spectral perturbations (ERSP). In relation to phasic pain, three components were described in earlier studies, including increased oscillations at low frequencies (1–10 Hz) between 150–400 ms post-stimulus, suppression of alpha and beta oscillations (8–29 Hz) between 300 and 1000 ms post-stimulus and increased gamma oscillations (30–100 Hz) between 150 and 350 ms post-stimulus^3,33–39^. These responses were shown to be modulated by both bottom-up and top-down processes^3^. Nevertheless, their functional significance in relation to pain perception remains unclear, although gamma oscillations seem to be more strongly associated with pain intensity in various contexts^3^. To date, how these brain responses may reflect the integration of information from concurrent painful stimuli remains unexplored. This may also reveal novel pain mechanisms that allow producing adapted brain responses and behaviors to protect body integrity optimally.

Here, in a set of four experiments, we examined the spinal and cerebral mechanisms of bilateral nociceptive input integration. We show that the RIII-reflex is facilitated when painful stimulation is applied to both lower limbs, along with increased pain perception. However, we show that with a short inter-stimulus delay, RIII-reflex and pain facilitation are abolished. Moreover, we show that concurrent stimulation of a lower limb and the contralateral upper limb facilitates the RIII-reflex, but not pain. This sensorimotor dissociation indicates that spinal inhibition and descending motor facilitation are integrated in the spinal cord. Lastly, we show that cerebral integration of bilateral nociceptive inputs was reflected in increased high-gamma oscillations in the dorsolateral prefrontal, anterior cingulate and sensorimotor cortex, providing a neural substrate for top-down cognitive regulation, descending motor regulation and pain regulation. Based on these findings, we propose that the brain and spinal cord can fine-tune nociceptive and pain responses depending on the location of nociceptive input sources, in order to provide adaptable responses.

## Results

### Nociceptive flexion reflex and pain perception

The RIII-reflex and pain thresholds as well as their correlation are reported in Table S1. For all experiments, RIII-reflex and pain thresholds were strongly and significantly associated (r = 0.62–0.81; p < 0.01). This is consistent with the idea that the RIII-reflex is produced by Aδ-fibre activation and with previous studies showing the strong relationship between RIII-reflex and pain thresholds^1,2^. Regarding the potential effect of sex hormone fluctuations on RIII-reflex threshold and modulation in femal participants^40–42^, groups were compared on their menstrual cycle day. The menstrual cycle day varied between Day 3 to Day 25, which covers all hormonal phases. Furthermore, the mean menstrual cycle day between groups was not significantly different (p = 0.7).

**Figure 1:**
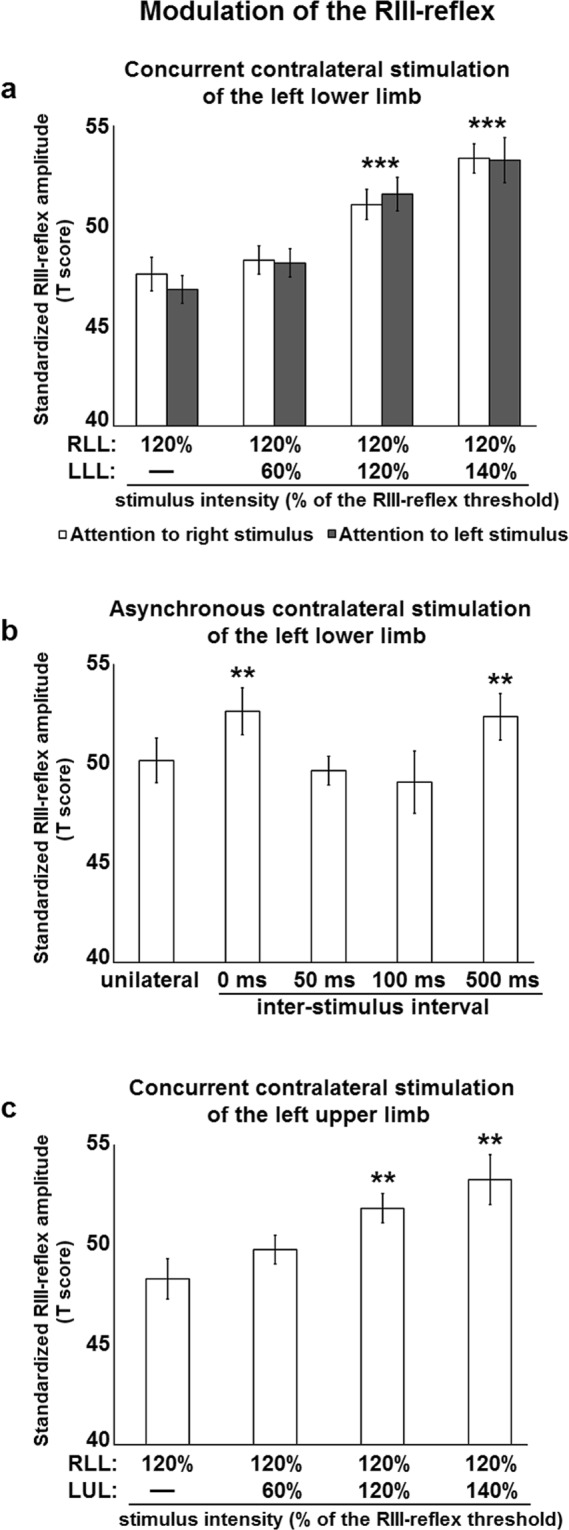
RIII-reflex modulation by contralateral stimulation. (**a**) The right RIII-reflex was facilitated by concurrent stimulation of the contralateral lower limb, regardless of attentional manipulation; main effect of intensity: F_3,72_ = 21.0, p < 0.001, *η*_*p*_^2^ = 0.47; no main effect of attention: F_1,24_ = 0.2, p = 0.67, *η*_*p*_^2^ = 0.01; no intensity-attention interaction: F_3,72_ = 0.3, p = 0.84, η_p_^2^ = 0.01. Significant differences for pairwise comparisons for the main effect of intensity are indicated with symbols (***p < 0.001, compared with unilateral stimulation). White and black histogram bars represent different conditions, in which the focus of attention was on the right or left lower limb, respectively; (**b**). The right RIII-reflex was modulated by contralateral stimulation of the lower limb; main effect: F_4,76_ = 2.8, p < 0.03, *η*_*p*_^2^ = 0.13. Significant differences for pairwise comparisons are indicated with symbols (**p < 0.01, compared with unilateral stimulation). (**c**) The right RIII-reflex was facilitated by concurrent stimulation of the contralateral upper limb; main effect: F_3,48_ = 4.0, p < 0.01; *η*_*p*_^2^ = 0.18. Significant differences for pairwise comparisons are indicated with symbols (**p < 0.01, compared with unilateral stimulation). RLL, right lower limb; LLL, left lower limb; LUL, left upper limb; Error bars represent standard error of the mean.

**Figure 2:**
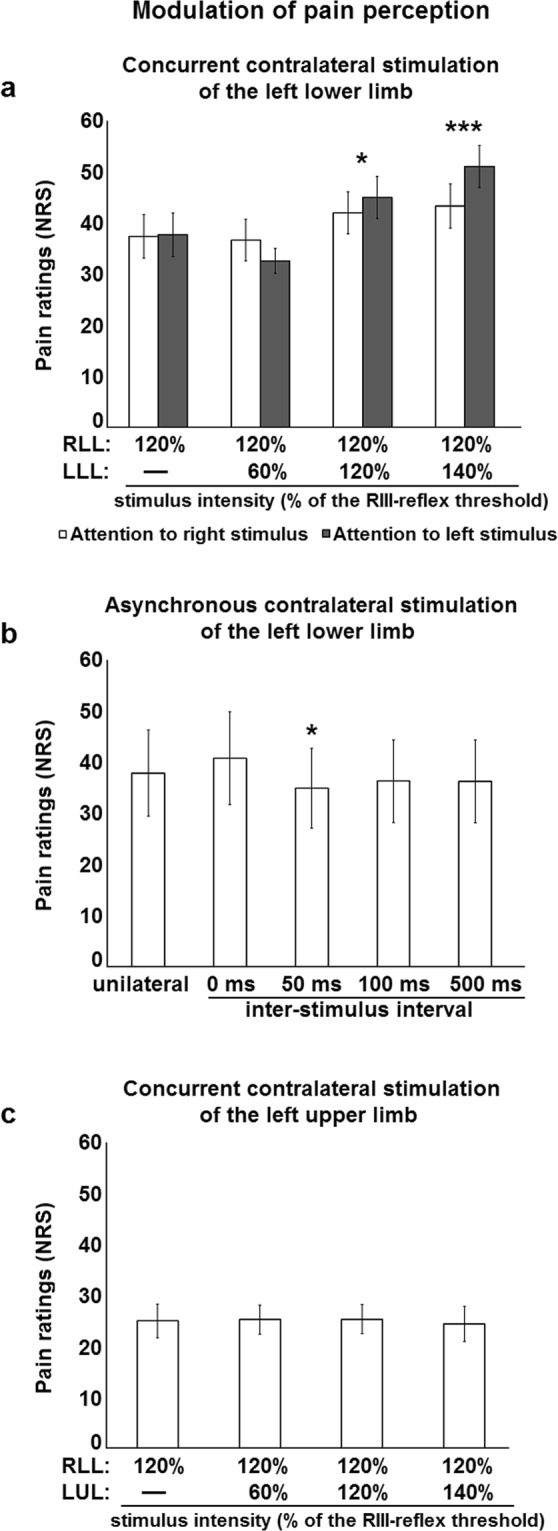
Modulation of pain perception by contralateral stimulation. (**a**) Right lower limb pain perception was increased by concurrent stimulation of the contralateral lower limb, regardless of attentional manipulation: main effect of intensity, F_3,72_ = 10.6, p < 0.001, *η*_*p*_^2^ = 0.31; no main effect of attention, F_1,24_ = 0.6, p = 0.44, *η*_*p*_^2^ = 0.03; no intensity-attention interaction, F_3,72_ = 2.5, p = 0.06, *η*_*p*_^2^ = 0.05. Significant differences for pairwise comparisons for the main effect of intensity are indicated with symbols (*p < 0.05, ***p < 0.001, compared with unilateral stimulation). White and black histogram bars represent different conditions, in which the focus of attention was on the right or left lower limb, respectively. (**b**) Right lower limb pain perception was modulated by contralateral stimulation of the lower limb; main effect: (F_4,76_ = 1.7, p = 0.17, *η*_*p*_^2^ = 0.08). Significant differences for pairwise comparisons are indicated with symbols (*p < 0.05, compared with unilateral stimulation). (**c**) Right lower limb pain perception was not modulated by concurrent stimulation of the contralateral upper limb: F_3,48_ = 0.2, p = 0.90, *η*_*p*_^2^ = 0.01. RLL, right lower limb; LLL, left lower limb; LUL, left upper limb; NRS, numerical rating scale. Error bars represent standard error of the mean.

To investigate the effects of contralateral inputs on the right RIII-reflex and pain perception, bilateral stimulation was applied concurrently on both lower limbs, in the innervation territory of the sural nerve. Compared with unilateral stimulation, the right RIII-reflex showed an intensity dependent facilitation by contralateral nociceptive inputs (p < 0.001), but not by contralateral non-nociceptive inputs (p = 0.09) (Fig. 1a). This is in contrast to the general rule on reflex movements evoked by limb stimulation^8,9^, but is consistent with peripheral excitatory projections to the contralateral spinal cord^28^. Similarly, right lower limb pain ratings showed an intensity dependent increase by contralateral nociceptive stimulation (p < 0.05) compared with unilateral stimulation, but contralateral non-nociceptive stimulation produced no effect (p = 0.3) (Fig. 2a). These effects on right RIII-reflex and pain perception were unaffected by spatial attention, as indicated by no significant difference between conditions with attention directed to the left or right stimulus (RIII-reflex: p = 0.7; pain: p = 0.4) (Figs 1a and 2a). This does not exclude all attentional effects, but at least indicates that RIII-reflex facilitation is not simply due to a shift of spatial attention.

To rule out the possible effect of posture on RIII-reflex facilitation, a second experiment was conducted with participants in a standing position, using the same procedures, except for the manipulation of attention that was omitted. Results from the first experiment were replicated (Supplementary Fig. 1a,b), indicating that contralateral nociceptive inputs can facilitate RIII-reflex and pain perception independently of posture.

To examine whether the expected spinal inhibition that could not be observed may depend on input timing, concurrent stimulation was compared with asynchronous stimulation of both lower limbs in a third experiment, by taking into account the latency of the crossed-extension reflex^27^. Consistent with results reported above, right RIII-reflex facilitation by concurrent contralateral nociceptive inputs was replicated (p < 0.01) (Fig. 1b). However, facilitation of the right RIII-reflex was abolished by contralateral nociceptive stimulation occurring 50 ms or 100 ms before right lower limb stimulation (both p’s = 0.11) (Fig. 1b). This is consistent with the latency of the crossed-extension reflex^27^ and with competing inhibitory processes. Hence, right RIII-reflex facilitation was observed when contralateral nociceptive stimulation occurred 500 ms before right lower limb stimulation (p < 0.01) (Fig. 1b), consistent with the long inter-stimulus interval and the lack of interaction between contralateral and ipsilateral inputs. This shows that spinal inhibition occurs when the synaptic timing of ipsilateral and contralateral inputs is in a time window that allows spinal neurons that underlie the crossed-extension reflex to interact with those of the RIII-reflex from the same limb. This also provides critical evidence indicating that competing excitatory and inhibitory inputs are integrated by the spinal cord when nociceptive stimulation occurs on both lower limbs, in order to produce adaptable reflexes. Similar results were observed for pain perception, where concurrent contralateral stimulation tended to increase right lower limb pain, although the effect was not significant (p = 0.3), while no facilitation was observed for asynchronous stimuli, with a slight but significant pain inhibition for the 50 ms inter-stimulus delay (p < 0.04) (Fig. 2b). This indicates that both the sensory and motor components of the RIII-reflex are modulated by contralateral nociceptive inputs.

To examine the contribution of supraspinal descending pathways to RIII-reflex and pain facilitation described above, concurrent stimulation was applied to the right lower limb and left upper limb, in a fourth experiment. Sensory inputs from the hand project to cervical spinal cord segments distant from lumbosacral segments receiving sural nerve afferent inputs^43^. This allows excluding segmental effects caused by contralateral inputs. In these conditions, the right RIII-reflex was still facilitated by contralateral nociceptive stimulation (p < 0.05) (Fig. 1c), but not by non-nociceptive stimulation (p = 0.24) (Fig. 1c). This indicates that concurrent contralateral nociceptive stimulation facilitates the right RIII-reflex when inputs are either segmental or heterosegmental, suggesting a contribution of descending pathways to RIII-reflex facilitation. In contrast, lower limb pain was not significantly modulated by either nociceptive or non-nociceptive contralateral stimulation (all p’s > 0.7) (Fig. 2c). This indicates that pain perception may be increased by contralateral nociceptive inputs, but only if they are segmental. This shows a sensorimotor dissociation with differential effects of contralateral inputs from lower and upper limbs, indicating that descending pathways that facilitate the RIII-reflex during bilateral stimulation act on the motor component of the reflex, while the sensory component remains unaffected.

### Brain activity

#### Event-related potentials

**Figure 3:**
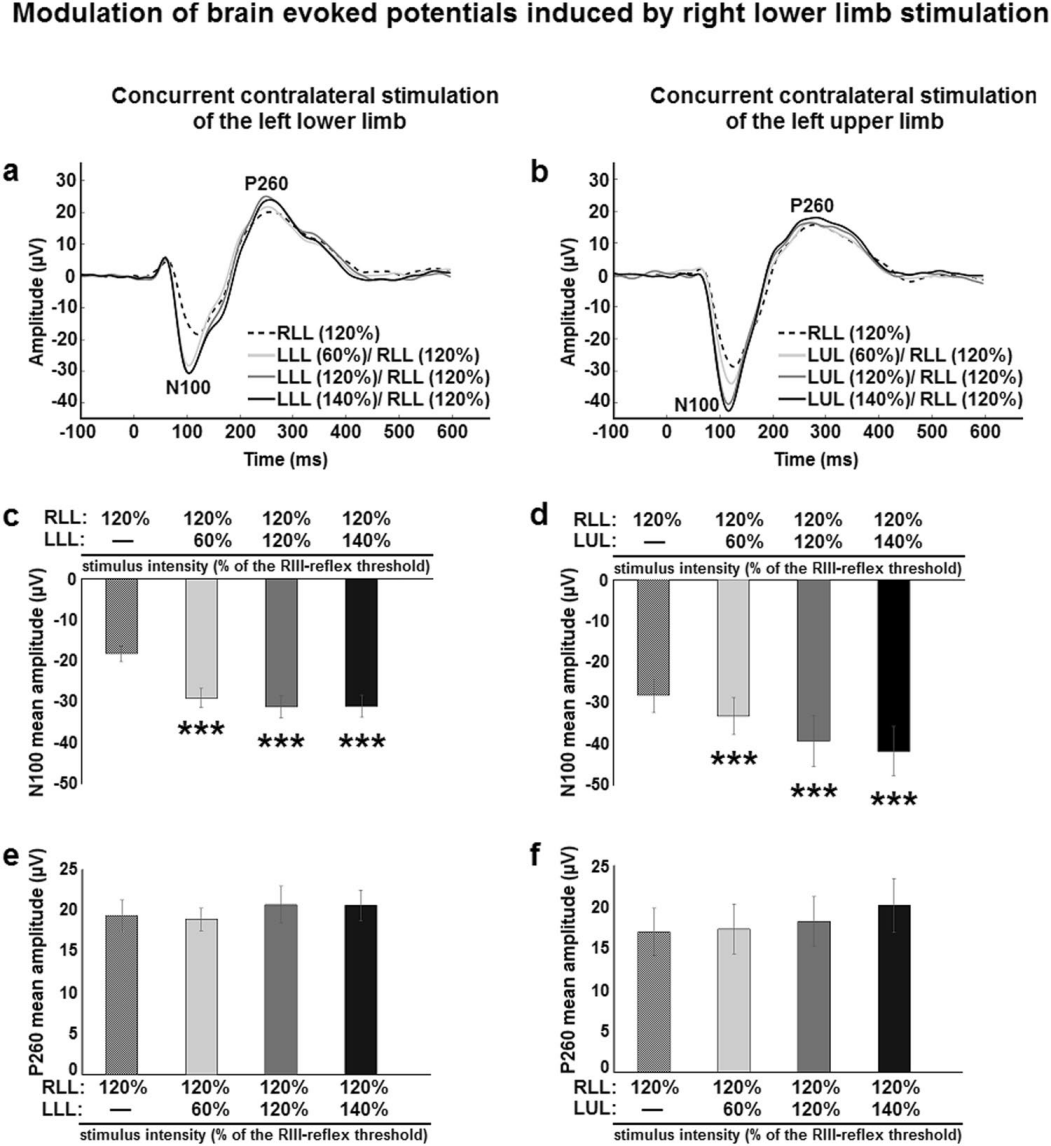
Facilitation of the brain activity by the concurrent contralateral stimulation.(**a,b**) Average ERPs (recorded at Cz) evoked by unilateral and bilateral stimulation. (**a**) Right and left lower limb stimulation. (**b**) Right lower limb and left upper limb stimulation. Dashed lines represent unilateral stimulation of the right lower limb at 120% of right RIII-reflex threshold. Gray, light black and dark black lines represent concurrent bilateral stimulation with contralateral stimulation intensity adjusted to 60%, 120% and 140% of left RIII-reflex (**a**) or pain (**b**) threshold, respectively; (**c**) Mean amplitude of the N100 evoked by right lower limb stimulation was increased by contralateral lower limb stimulation; main effect: F_3,72_ = 88.3, p < 0.001, *η*_*p*_^2^ = 0.66. Significant differences for pairwise comparisons are indicated with symbols (***p < 0.001, compared with unilateral stimulation). (**d**) Mean amplitude of the N100 evoked by right lower limb stimulation was increased by contralateral upper limb stimulation; main effect: F_3,48_ = 16.1, p < 0.001, *η*_*p*_^2^ = 0.5; Significant differences for pairwise comparisons are indicated with symbols (***p < 0.001, compared with unilateral stimulation). (**e**) P260 mean amplitude was not modulated by contralateral lower limb stimulation; main effect: F_3,72_ = 1.8, p = 0.15, *η*_*p*_^*2*^ = 0.04. (**f**) Mean amplitude of the P260 evoked by right lower limb stimulation was not modulated by contralateral upper limb stimulation; main effect: F_3,48_ = 2.2, p = 0.09, *η*_*p*_^*2*^ = 0.12. Dashed columns represent unilateral stimulation of the right lower limb at 120% of right RIII-reflex threshold. Gray, light black and dark black columns represent concurrent bilateral stimulation with contralateral stimulation intensity adjusted to 60%, 120% and 140% of left RIII-reflex threshold, respectively; RLL, right lower limb; LLL, left lower limb; LUL, left upper limb. Error bars represent standard error of the mean.

Electrical stimulation of the sural nerve produced robust ERPs, with notable N100 and P260 components for all participants in all conditions (Fig. 3a,b). Mean amplitude and peak latencies for both components are reported in Table 1. In order to examine whether the RIII-reflex and pain modulation by contralateral stimulation is reflected in differential ERPs, the mean amplitude and peak latency of the N100 and P260 were compared between conditions.

**Table 1:** Mean amplitude and peak latency of N100 and P260 (mean ± SEM).

Right lower limb	Stimulus intensity			
	120%	120%	120%	120%
Left lower limb	—	60%	120%	140%
N100				
*Amplitude (µV)*	−18.3 ± 1.9	−29.1 ± 2.4***	−31.3 ± 2.7***	−31.1 ± 2.7***
*Latency (ms)*	112.8 ± 1.8	105.8 ± 2.0***	105.8 ± 1.8***	106.7 ± 1.9***
P260				
*Amplitude (µV)*	19.4 ± 9.3	18.9 ± 6.7	20.7 ± 10.8	20.6 ± 9.2
*Latency (ms)*	294.9 ± 4.9	291.5 ± 4.3	288.3 ± 3.4	288.3 ± 3.6
**Right lower limb**	**120%**	**120%**	**120%**	**120%**
**Left upper limb**	—	**60%**	**120%**	**140%**
N100				
*Amplitude (µV)*	−28.1 ± 4.3	−33.2 ± 4.5***	−39.3 ± 6.2***	−41.7 ± 6***
*Latency (ms)*	114.6 ± 2.1	114.1 ± 1.9	114.4 ± 1.6	112.5 ± 2.0
P260				
*Amplitude (µV)*	17.0 ± 2.9	17.3 ± 3.0	18.3 ± 3.0	20.2 ± 3.2
*Latency (ms)*	301.6 ± 5.7	297.8 ± 5.2	309.5 ± 7.3	309.6 ± 7.0

***p < 0.001, compared with unilateral stimulation.

The mean amplitude of the N100 evoked by right lower limb stimulation was significantly increased by inputs from the contralateral lower limb (p < 0.001) (Fig. 3c) and from the contralateral upper limb (p < 0.001) (Fig. 3d). This was true for both painful and non-painful stimuli (all p’s < 0.001). As for peak latency of the N100 evoked by right lower limb stimulation, it was significantly decreased by inputs from the contralateral lower limb (all p’s < 0.001) (Table 1). However, this peak latency decrease was not observed when the contralateral upper limb was stimulated concurrently (p < 0.7) (Table 1), consistent with pain modulation but not RIII-reflex modulation. This suggests that the N100 is more closely related to sensory rather than motor processes, in accordance with the activation of pain-related processes in the supplementary somatosensory area, in which the N100 generator is located^44^. Increased amplitude of the N100 during concurrent bilateral stimulation also indicates that stimulus saliency increased, consistent with the activation of more nociceptive fibers and the sensory representation of a unique coherent percept of increased threatfullness.

In contrast to these effects, the mean amplitude and peak latency of the P260 evoked by right lower limb stimulation were not significantly modulated by inputs from either the contralateral lower limb (p = 0.5 and p = 0.3, respectively) or the contralateral upper limb (p = 0.1 and p = 0.3, respectively) (Fig. 3e,f; Table 1). This indicates that cerebral integration of sensory information occurs at early steps of pain-related information processing.

#### Event-related spectral perturbations

**Figure 4:**
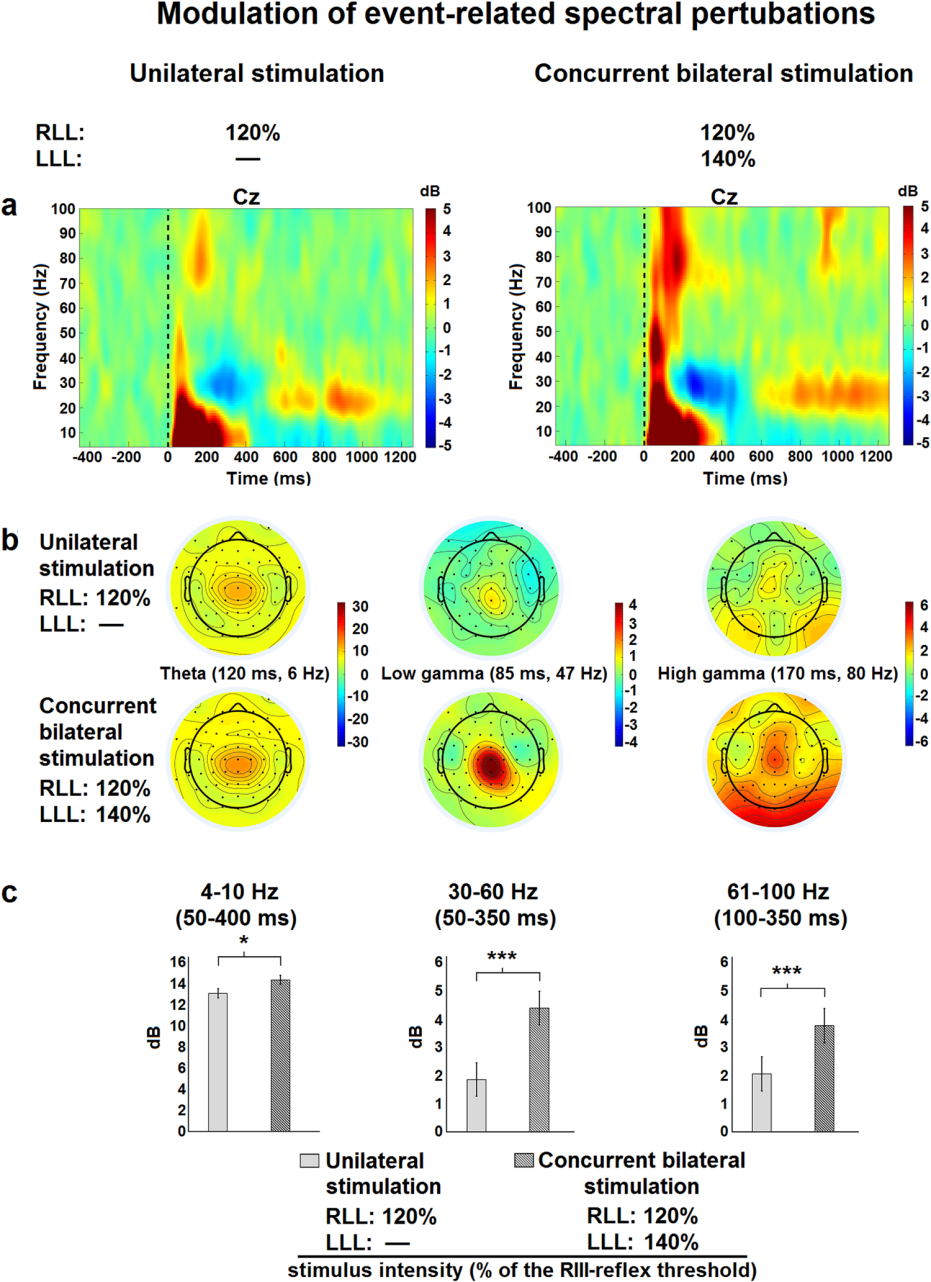
Modulation of event-related spectral pertubations. (**a**) Mean stimulus-locked event-related spectral pertubations at electrode Cz. The dashed line indicates the stimulus onset. Oscillation power is presented in dB relative to a pre-stimulus baseline (−400 ms to −100 ms). Positive and negative power changes are represented by red and blue colors, respectively. (**b**) Topographic representation of brain activity for time-frequency points representing the maximal activity at frequency ranges that were statistically different between conditions acording to top 20% approach (theta-, low gamma- and high gamma-bands). Positive and negative power changes are represented by red and blue colors, respectively. (**c**) Power values in dB. Input from the left lower limb caused a significant increase in theta (t (19) = 2.5, p = 0.02), low gamma (t (19) = 6.8, p = 0.001) and high gamma frequency bands (t (19) = 4.2, p = 0.001). RLL, right lower limb; LLL, left lower limb.

To further examine brain processes underlying the cerebral integration of bilateral nociceptive inputs reported above, ERSPs were compared between unilateral and bilateral lower limb stimulation, for which significant differences were observed. In the first analysis based on regions of interests in the time-frequency map from Cz signal (Fig. 4a), the mean power of ERSPs evoked by right lower limb stimulation was significantly increased by nociceptive inputs from the contralateral lower limb for 4–10 Hz oscillations (p < 0.02), low gamma oscillations (30–60 Hz) (p < 0.001) and high gamma oscillations (61–100 Hz) (p < 0.001) (Fig. 4b,c), while no significant change in event-related synchronization or desynchronization was observed for the 8–29 Hz oscillations (p = 0.22 and p = 0.13, respectively). These effects were centered at Cz and extended to electrodes over the sensorimotor cortex (Fig. 4b).

**Figure 5:**
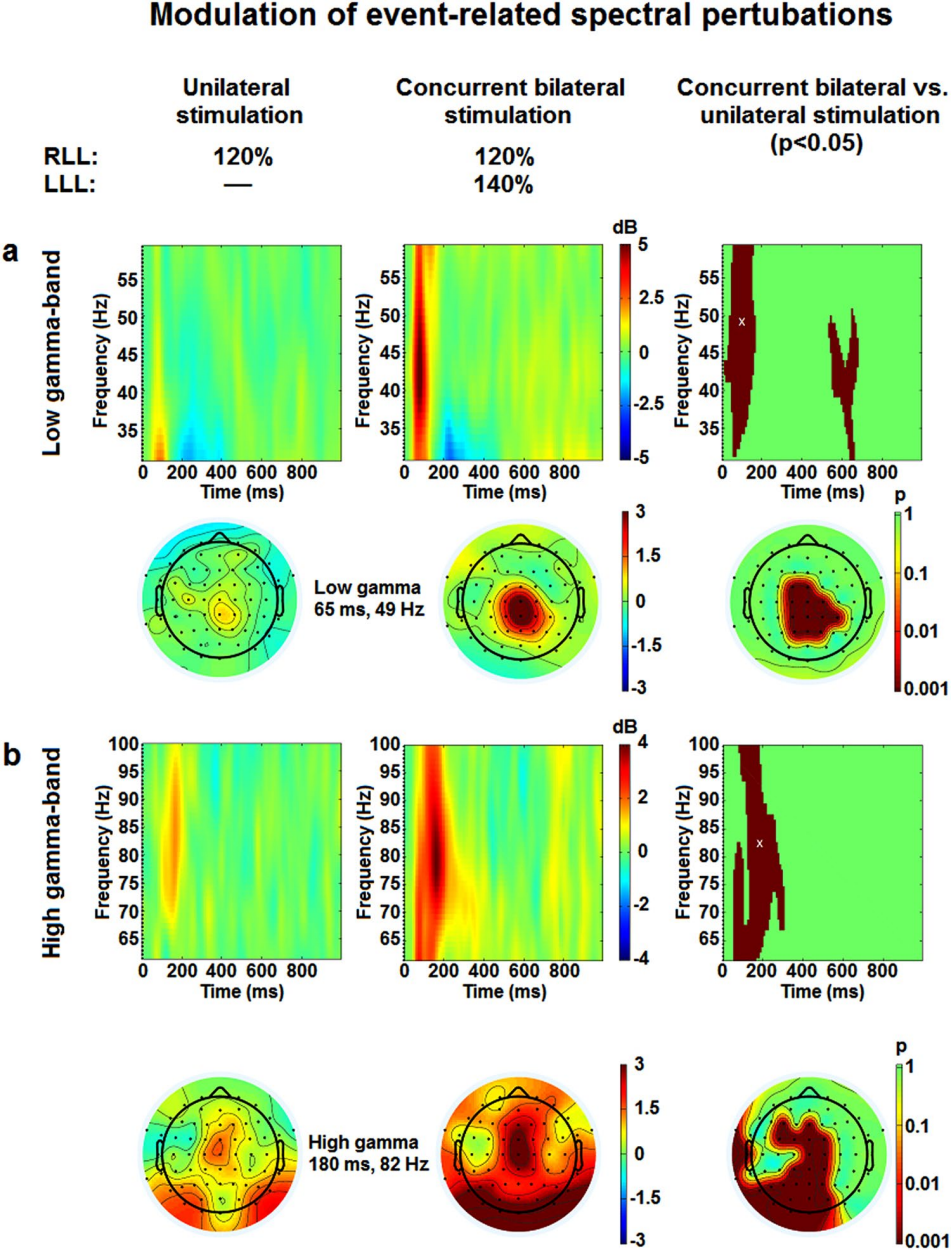
Modulation of event-related spectral pertubations by concurrent contralateral input from the left lower limb: permutation analysis with cluster correction for multiple comparisons. (**a**,**b**) Top, Mean stimulus-locked event-related spectral pertubations to unilateral and bilateral stimulation at electrode Cz for low gamma- and high gama-bands, respectively. Responses are displayed in dB relative to a pre-stimulus baseline (−400 ms to −100 ms). Increase in power by contralateral input for both low gamma- and high gamma-bands (p < 0.05). (**a**,**b**) Bottom, The difference in brain activity was distributed over sensory-motor area centering at Cz for both low gamma- and high gamma-bands. RLL, right lower limb; LLL, left lower limb.

In the second analysis, using permutation with cluster-correction (p < 0.05) without temporal and spectral prior, we observed that nociceptive inputs from the contralateral lower limb significantly increased mean power of low and high gamma oscillations evoked by right lower limb stimulation (Fig. 5a,b, top). This confirms results from the first analysis and also extends these findings by showing that effects occurred between 0 and 200 ms and between 500 and 650 ms for low gamma oscillations, while the effects for high gamma oscillations occurred between 50 and 300 ms. In both cases, effects were centered at Cz and extended to electrodes over the sensorimotor cortex, with a larger scalp distribution for high gamma oscillations (Fig. 5a,b, bottom). The early effects are consistent with increased pain ratings and N100 amplitude reported above and support the idea that the cerebral integration of bilateral nociceptive inputs is reflected by increased cerebral activity and changes in activity pattern and scalp distribution. Effects observed at longer latencies also indicate that cerebral integration occurs at later steps of sensorimotor information processing, suggesting that processes involved in regulation of pain and motor responses are activated.

#### Source estimation

**Figure 6:**
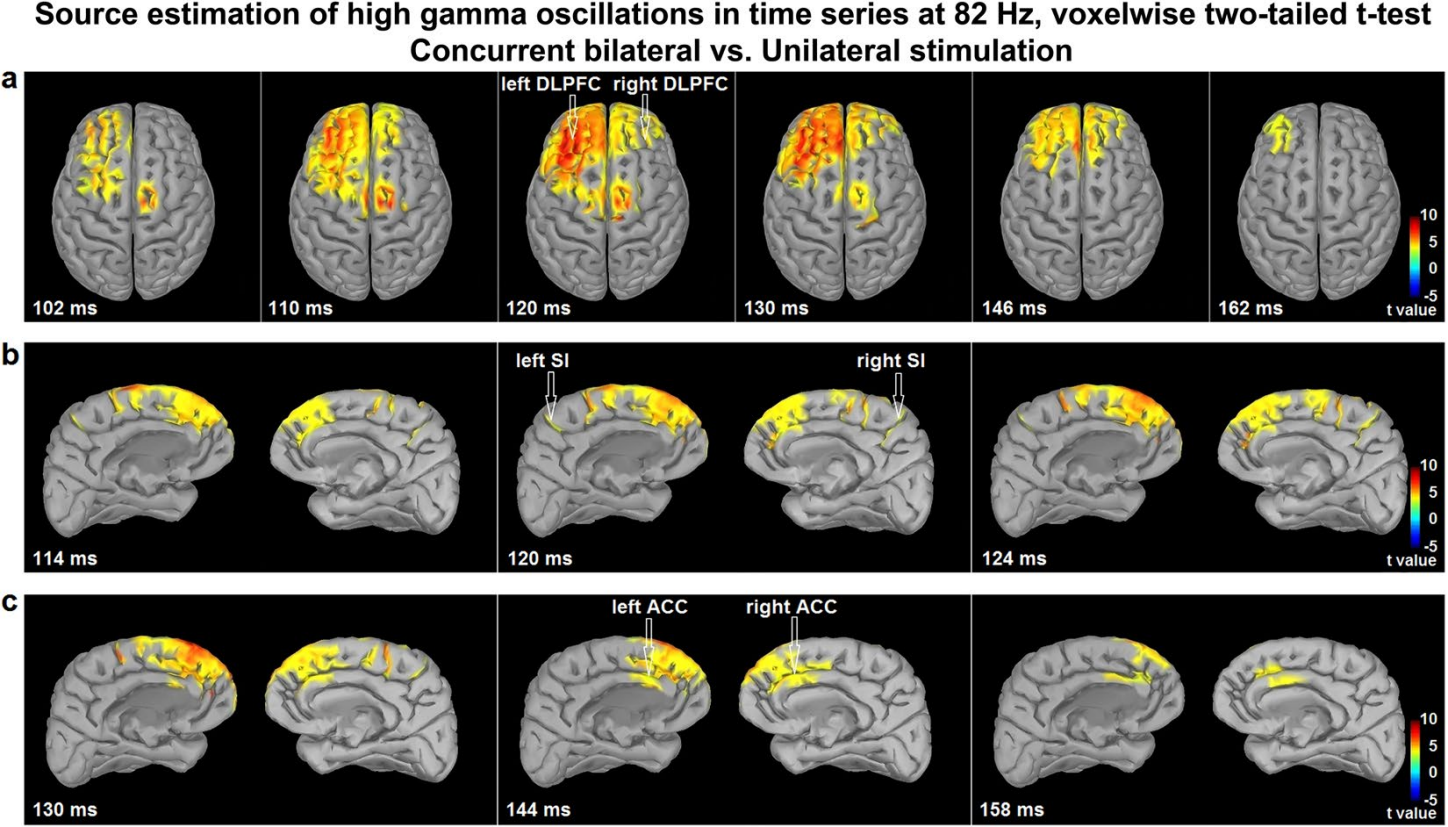
Source estimation of increased high gamma oscillations during bilateral stimulation. Time courses of high gamma oscillations induced by bilateral stimulation were compared to those induced by unilateral stimulation, using a voxelwise two-tailed paired t-test on time-frequency source space at 82 Hz. The contrast revealed sources of increased high gamma oscillations in the following regions: (**a**) Lateral prefrontal cortex bilaterally, maximally over the left DLPFC (102–162 ms). Pre-motor cortex bilaterally (102–130 ms). (**b**) Foot region of primary somatosensory cortex (SI) bilaterally (114–124 ms). Medial prefrontal cortex bilaterally (102–160 ms). (**c**) Posterior part of ACC extending to the anterior MCC and cingulomotor area bilaterally (130–158 ms). Positive and negative relationships are depicted by warm and cold colors, respectively. Whole-brain t-maps were thresholded at p < 0.05, false discovery rate corrected for the whole brain.

The significance of brain oscillations evoked by nociceptive stimulation and pain is still limited. However, previous studies provided compelling evidence that high-gamma oscillations are closely related to pain perception^3^. To examine the sources of high-gamma power increase in the bilateral condition, source estimation was calculated for the contrast between concurrent bilateral and unilateral stimulation. The analysis revealed that statistically significant foci of high gamma power increase were located in the bilateral lateral prefrontal cortex (maximal over left DLPFC), bilateral medial prefrontal cortex, bilateral foot region of primary somatosensory cortex (SI) and pre-motor regions (Fig. 6a,b). These effects were observed from 102 ms to 162 ms post-stimulation, mostly at 70–90 Hz, peaking at 120 ms and 82 Hz (Fig. 6a,b and Supplementary Video 1). Moreover, additional foci were observed in the posterior part of ACC extending to the anterior MCC and cingulomotor area bilaterally. The latter effect lasted from 130 ms to 158 ms peaking at 82 Hz (Fig. 6c and Supplementary Video 1). MNI coordinates (x, y, z) of t-value peaks included the left DLPFC (−46, 38, 8), the right anterior prefrontal cortex (31, 54, 26), the medial prefrontal cortex on the left (−2, 45, 36) and right (1, 43, 40), SI on the left (−6, −42, 79) and right (−2, −42, 72), the pre-motor cortex on the left (−3, 7, 71) and right (4, 9, 64) as well as the posterior ACC/anterior MCC on the left (−1, 27, 19) and right (0, 32, 23). These results are consistent with the activation of cerebral processes related to top-down cognitive regulation, descending motor regulation and pain regulation^6,45–49^.

## Discussion

Facilitation of the RIII-reflex by concurrent contralateral stimulation observed in the current study contrasts with the expected inhibitory effects of bilateral lower limb stimulation due to the crossed-extention reflex and reciprocal innervation. Facilitation of the RIII-reflex by contralateral nociceptive inputs provides evidence for spinal integration for conditions in which both limbs should be withdrawn simultaneously. Although contralateral facilitatory projections in the spinal cord cannot be excluded^28,29^, the present results support the involvement of descending facilitatory pathways from cortical areas^50^, which override spinal inhibition during concurrent stimulation. Indeed, facilitation of the RIII-reflex was abolished when nociceptive activity from the contralateral lower limb was evoked 50 ms or 100 ms earlier, showing competition between facilitation and inhibition.

In line with these results, gamma oscillation power was increased in the cingulate cortex, sensorimotor areas and DLPFC during concurrent bilateral stimulation. Increased gamma power by painful stimuli is consistent with previous studies^33,34,51^. The present results extend these findings and show that pain-related activity in these regions is related to the regulation of pain and spinal nociceptive reflexes. Accordingly, the cingulate cortex is well-known for its involvement in pain affect as well as cognitive and motor processes^6,45,49,52–58^. Hence, top-down regulation through ACC modulates activity in the sensory, motor, and association areas^47,48^ and rostral ACC is one of the key regions of descending pain modulation^59–61^. Moreover, the motor areas of ACC^62,63^, which receive inputs from the spinothalamic tract^64^, primary motor cortex, premotor cortex and supplementary motor area^65,66^, give rise to direct corticospinal projections^46,65^. Regarding the DLPFC, it was reported that dividing attention between simultaneous auditory and visual events leads to its activation, which is not the case when attention is directed selectively towards one of these events^67,68^. These findings show that the DLPFC is crucial to support attention during processing of multiple concurrent stimuli. In addition, reciprocal cortico-cortical connections between DLPFC and the ACC support their contribution to cognitive control^46^. Therefore, gamma synchronization at similar latency in ACC and DLPFC during bilateral stimulation is coherent with increased cognitive control to regulate pain-related responses.

It should be mentioned that A-beta fibres may contribute to the RIII-reflex measured in this study. Indeed, A-beta fibres activated by cutaneous stimulation can produce a lower limb flexion reflex through a transcortical pathway^69^. However, this seems unlikely in the present study based on findings reported previously^1,25^ showing that A-delta fibres must be activated by the electrical stimulus to produce the RIII-reflex, while maximal A-beta activation only produces a cutaneomuscular reflex and a tactile sensation. A lidocaine block of A-delta fibres also abolishes the RIII-reflex^25^, excluding the contribution of other fibre groups to the RIII-reflex. Furthermore, it should be mentioned that if any interaction occurs between A-beta fibres and the RIII-reflex, A-beta fibres would suppress the RIII-reflex and not produce facilitation^24^, which is consistent with the facilitation of the RIII-reflex by an ischemic block of A-beta fibres^25^. In addition, this does not exclude that C-fibres may facilitate the nociceptive withdrawal reflex, as shown by applying a concomitant heat stimulus^70^ or capsaicin^71^. But these conditions were not used in the present experimental design and therefore we can clearly state that the measured response depends entirely on A-delta fibres.

## Conclusion

The present results indicate that during concurrent bilateral lower limb stimulation, the spinal cord integrates spinal inhibitory and descending motor facilitatory inputs, resulting in facilitation of the nociceptive flexion reflex and bilateral flexion. Consistent with this, cerebral integration of bilateral nociceptive inputs was reflected in increased high-gamma oscillation power in the dorsolateral prefrontal, anterior cingulate and sensorimotor cortex, in accordance with their involvement in top-down cognitive regulation, descending motor regulation and pain regulation. Based on these findings, we propose that the brain and spinal cord can fine-tune nociceptive and pain responses when nociceptive inputs arise from multiple sources, in order to allow adaptable responses.

## Material and Methods

### Ethics approval

All experimental procedures conformed to the standards set by the latest revision of the Declaration of Helsinki and were approved by the Research Ethics Board of Université du Québec à Trois-Rivières. All participants gave written informed consent, acknowledging their right to withdraw from the experiment without prejudice and received compensation for their travel expenses, time and commitment. The study consisted in four experiments of about 90 minutes each, including preparation, the determination of thresholds (RIII-reflex and pain) and evaluation of RIII-reflex, pain and brain activity modulation.

### Study participants

Participants were recruited by advertisement on the campus of Université du Québec à Trois-Rivières. Participants were included in the study if they were between 18 and 50 years old and were excluded if they had taken any medication within 2 weeks prior to the experiment, if they had any history of acute or chronic pain, including episodic primary headaches, acute or chronic illness, or a diagnosed psychiatric disorder. Different participants were recruited for the four experiments. Female participants were tested randomly across their menstrual cycle and their menstrual cycle day was noted to confirm that no difference was observed between experiments, considering that the hormonal level or phase may alter RIII-reflex responses and their modulation^40–42^.

### Experimental design

The four experiments of this study relied on a within-subject design and included a total of 80 healthy volunteers. Supplementary Fig. 2 shows the experimental design for each of the four experiments. In all four experiments, the right sural nerve was stimulated at an individually adjusted intensity of 120% of the right RIII-reflex threshold. In *Experiment 1* (N = 25, 15 women and 10 men; age range 19–30 years; mean ± SD: 24.4 ± 3.1 years), contralateral stimulation was applied to the left sural nerve, adjusted individually to 60% (non-nociceptive/tactile), 120% or 140% (nociceptive/painful) of the left RIII-reflex threshold, while participants were in a supine position. Participants were instructed to attend either the right or left stimulus to test the potential effect of selective attention. In *Experiment* 2 (N = 18, 8 women and 10 men; age range 21–44 years; mean ± SD: 26 ± 6.0 years), the same procedures were performed, but participants were in a standing position, in order to exclude postural effects. Manipulation of attention was not performed for this experiment based on the lack of effect in the first experiment. In *Experiment 3* (N = 20, 11 women and 9 men; age range 20–38 years; mean ± SD: 24.5 ± 5.6 years), electrical stimulation was applied either unilaterally, bilaterally and concurrently or bilaterally and asynchronously with participants in a supine position, based on the lack of postural effects observed in *Experiment* 2. When asynchronous, contralateral stimulation of the left sural nerve was applied earlier with an inter-stimulus interval of 50 ms, 100 ms or 500 ms. In this experiment, stimulus intensity remained constant at 120% of the RIII-reflex thresholds for both limbs. In *Experiment 4* (N = 17, 6 women and 11 men; age range 21–37 years; mean ± SD: 24 ± 4.0 years), the same procedures as in *Experiment 1* were performed without manipulating attention, but contralateral stimulation was applied to the left upper limb at 60% (non-nociceptive/tactile), 120% or 140% (nociceptive/painful) of pain threshold. In the supine position, participants lied down on their back with ankle and knee flexion of approximatly 90° and 120°, respectively. In all four experiments, each condition included a series of 21 electrical stimuli delivered with an inter-stimulus interval of 6–12 seconds varying pseudo-randomly, for a total of 84 to 168 stimuli depending on the experiment. The order of conditions was always counterbalanced to avoid sequence order effects.

### Electrical stimulation of the sural nerve

Transcutaneous electrical stimulation (trains of 10 × 1-ms pulses at 333 Hz) was delivered with an isolated DS7A constant current stimulator (Digitimer Ltd., Welwyn Garden City, Hertfordshire, UK) triggered by a Grass S88 train generator (Grass Medical Instruments, Quincy, MA, USA) and controlled by computer with a stimulus presentation program (E-Prime2, Psychology Software Tools, Sharpsburg, PA, USA). Degreased skin over the retromalleolar path of the sural nerve was stimulated by a pair of custom-made surface electrodes (1 cm^2^; 2 cm inter-electrode distance). The right and left RIII-reflex thresholds were determined separately using the staircase method including 4 series of stimuli of increasing and decreasing intensity, as in our previous studies^72,73^. Stimulus intensity was then adjusted individually to 60%, 120% or 140% of RIII-reflex threshold depending on the experimental condition. Pain threshold was also determined using the staircase method and was defined as the lowest stimulus intensity evoking pain. Stimuli were then adjusted individually to 60%, 120% or 140% of pain threshold depending on the experimental condition.

### RIII-reflex measure and analyses

Electromyography (EMG) of the short head of the right and left biceps femoris was recorded with a pair of surface electrodes (EL-508, Biopac Systems, Inc., Goleta, CA, USA). It was amplified 1,000 times, band pass filtered (10–500 Hz), sampled at 1,000 Hz (BiopacSystems, Inc.) and stored on a personal computer for off-line analyses. Right RIII-reflex data were analyzed with Acknowledge 4.1.1 software. Raw EMG recordings were full-wave rectified and the resulting signal was used to quantify the amplitude of the RIII-reflex to each shock by extracting the integral value between 90–180 ms after the stimulus onset, as in our previous studies^72,73^. This amplitude was normalized within each subject for each shock using T scores, mean-centered at 50. The mean of the 21 responses was then calculated for each condition to make comparisons.

### Pain ratings

A visual analog scale was shown to participants on a computer monitor to prompt the evaluation of pain induced by electrical stimulation of the right sural nerve. It was placed horizontally and included the verbal anchors at the extremities, “no pain” and “worst pain imaginable”. Employing these scales, participants were instructed to rate pain verbally, with numbers between 0 and 100 corresponding to the left and right anchors of the scale. Participants were prompted to rate shock pain after each stimulation of the right sural nerve.

### Electroencephalographic recording

In experiments 1 and 4, electroencephalogram (EEG) was recorded at Fz, Cz, C3, C4 and Pz using a monopolar montage with a right ear lobe reference and FPz as a ground (Electro-cap International Inc., Eaton, OH, USA). Electro-oculographic activity was recorded using a pair of electrodes placed above and below the right eye with a ground placed on the forehead. Electrode impedance was kept below 10 kΩ. Electroencephalographic and electro-oculographic signals were filtered with a 0.1–35 Hz bandpass (EEG100C, EOG100C; Biopac Systems) and sampled at 250 Hz (MP150 with ACQKNOWLEDGE software v4.1.1.; Biopac Systems) for offline analyses.

In experiment 3, to conduct time-frequency and source estimation analyses, EEG was recorded by means of a 64-channel BrainAmp amplifier, using active Ag–AgCl electrodes which were mounted on an actiCAP in an International 10–20 System montage (Brain Products, Gilching, Germany). The electrodes were referenced to the nose-reference, with a ground electrode placed on the forehead. Electro-oculographic activity was recorded using a pair of electrodes placed at the suborbital ridge (vertical electro-oculogram, vEOG) and at the external ocular canthus (horizontal electro-oculogram, hEOG) of the right eye. Electrode impedance was kept below 10 kΩ. Electroencephalographic and electro-oculographic signals were filtered with a 0.01–100 Hz bandpass and sampled at 500 Hz for offline analyses.

### Event-related potentials analysis

EEG data were analyzed in MATLAB (Mathworks, Nattick, MA, USA) environment using EEGLAB version 14_1_1b^74^. Data were filtered offline using a FIR filter with the lower edge of the frequency passband at 0.1 Hz and a higher edge of the frequency passband at 30 Hz. Data was screened for extreme values, as well as for infrequent and unstereotyped artifacts using the inbuilt probability function (pop_jointprob) with a threshold of 3 SD^75^. For further artifact attenuation, Infomax independent component analysis (ICA) was applied. Artifacts were identified using the EEGLAB-Runica function, and ICs found to reflect blinks, lateral eye movements, muscle-related and cardiac artifacts were removed from the data. Following ICA-based artifact attenuation, ERPs were time-locked to the right sural nerve stimulation, baseline-corrected between −100 ms and 0 prior to the right sural nerve stimulation and averaged for each condition. The amplitude of the N100 and P260 components was quantified using the mean amplitude between fixed latencies (N100: 90–120 ms post-stimulus; P260: 280–350 ms post-stimulus). The P45 and N150 components were not clearly observed in all conditions and all participants. They are therefore not reported.

### Time-frequency analysis

Time-frequency analysis was also performed in MATLAB (Mathworks, Nattick, MA, USA) environment using EEGLAB version 14_1_1b^74^. EEG was filtered offline using a FIR band pass filter (1–100 Hz). As described above for ERPs, data were screened for extreme values, as well as for infrequent and unstereotyped artifacts and ICA analysis was applied to remove the reminder of artifacts. Data were segmented into stimulus-locked epochs from −1600 to 2600 ms, with time 0 corresponding to the onset of electrical stimulation. A Morlet wavelet convolution^76^ was computed using the channel time-frequency options available on EEGLAB 14_1_1b^74^. Two hundred time points were generated and 100 linearly spaced frequencies were computed from 1 to 100 Hz. Variable cycles were used for low and high frequencies, with 3 cycles for lowest frequencies and up to 15 cycles for highest frequencies. This variable cycle allows the wavelet convolution method to provide a better frequency resolution at lower frequencies and a better temporal resolution at higher frequencies^77^. ERSPs^78,79^ were computed in decibels relative to the −400 to −100 ms baseline as shown below, where ERSPt_f_ represents ERSP data in decibels for each time and frequency point:

$$ERSP{t}_{f}=10\ast \,\mathrm{log}\,10\,(\frac{{\rm{mean}}\,{\rm{baseline}}\,{\rm{power}}\,{\rm{at}}\,{\rm{each}}\,{\rm{frequency}}}{{\rm{power}}\,{\rm{at}}\,{\rm{each}}\,{\rm{time}}\,{\rm{point}}\,{\rm{and}}\,{\rm{each}}\,{\rm{frequency}}})$$

Based on previous studies^3^, mean power in the four time-frequency maps from Cz signal were extracted in pre-determined regions of interest (*time* × *frequency*) from 4 to 10 Hz between 50 and 400 ms, from 8 to 29 Hz between 250 and 1000 ms, from 30 to 60 Hz between 50 and 350 ms, and from 61 to 100 Hz between 100 and 350 ms. The gamma band was separated into a low and high frequency components^80^. The ERSPt_f_ value for each time-frequency point included in the regions of interest was calculated for each subject. A mean ERSPt_f_ value was then obtained for each subject over the regions of interest by averaging the values with the 20% highest power (for power increase relative to the baseline) or 20% lowest power (for power decreases relative to the baseline). This procedure has been used in previous studies for time-frequency analysis^38,81–83^ and presents the main advantage of being able to select wide regions of interests, thus taking into account the variability across subjects, while reducing the regression to the mean problem with near-zero values. Distribution of the brain activity was illustrated for time-frequency points representing the maximal activity at frequency ranges that were statistically different between conditions acording to top 20% approach. For each participant, the time-frequency data were averaged across all trials per condition. The grand average time-frequency maps for the group were obtained by averaging data across all subjects for each condition.

For the second analysis, a data-driven approach was used to examine differences across all time-frequency points between 0 and 1000 ms for signal recorded at Cz electrode. Specific frequency bands were defined as follows: theta (4 to 7 Hz), alpha (8 to 12 Hz), beta (13 to 29 Hz), low gamma (30 to 60 Hz) and high gamma (61 to 100 Hz). For each frequency band, differences between bilateral and unilateral conditions were computed as *t*-values for each time-frequency point. Monte Carlo permutation analysis with 2000 permutations was used to create a permutation distribution, testing the null hypothesis that the data from both conditions are drawn from similar probability distributions. Within a permutation distribution, temporally and spectrally adjacent *t*-values with similar magnitude and sign were clustered. All *t*-values comprised in a cluster were summed and the largest cluster-level statistic was used as a comparator for all the other smaller clusters^84^. Time-frequency clusters were then explored across electrodes. Within a given time-frequency cluster, the time and frequency with the highest *t*-value was selected for time and frequency plotting across all electrodes. The grand average time-frequency map for the group was also obtained for each condition by averaging data across subjects.

### Source estimation

The freely available source estimation package implemented in Brainstorm software was used to estimate the cortical sources of high-gamma oscillations in experiment 3^85^. The forward model was calculated using the Open-MEEG Boundary Element Method^86^ on the cortical surface of a template MNI brain (colin27 atlas) with 1 mm resolution. A noise covariance matrix was estimated from the preprocessed EEG data. Cortical source activation was calculated with a constrained inverse model of EEG sources using the weighted minimum norm current estimation^87^ and mapped to a distributed source model consisting of 15.002 elementary current dipoles. We then computed time-frequency decomposition on source time series for each trial using the Morlet transform from 60 Hz to 100 Hz in 1 Hz steps. The resulting maps across trials were averaged for each subject. Consistent with the ERP analysis, the source analysis was focused on the time window of the N100 component (90–120 ms). Two-tailed t-test between conditions (experiment 3: concurrent bilatral stimulation of both lower limbs vs. unilateral stimulation of the right lower limb) was applied to each point in space to identify statistically significant voxels (frequency range 70–90 Hz, time window 0–200 ms). In order to minimize the possibility of erroneous results, we present source estimations if the statistically significant differences at the source level survive the FDR-based multiple comparison correction. Since we used relatively small number of electrodes (n = 64) and a standard template for head model calculation, the spatial precision of the source estimations is limited.

### Statistical analyses

Data analysis was conducted using Statistica v13.1 (Dell Inc., Tulsa, OK, USA). All results are expressed as mean ± SEM and statistical threshold was set at p ≤ 0.05 (two-tailed). Modulation of the RIII-reflex, pain, ERP amplitude and ERSPt_f_ within each experiment was evaluated by repeated-measures ANOVA with CONDITION (different blocks of 21 stimuli) as a repeated factor. For *Experiment 1*, ATTENTION (left or right stimulus) was added as a second repeated factor. Predetermined planned contrasts were used to test a priori hypotheses. Effect sizes are reported based on partial eta-squared (η_p_^2^).

## Supplementary information

Supplementary Video file

Supplementary Material

## Data Availability

Data are available from the corresponding author (M.P.) upon reasonable request.
