# Supplementary material for: Integration of bilateral nociceptive inputs tunes spinal and cerebral responses
Source: Sci Rep. 2019 May 9;9:7143. doi: 10.1038/s41598-019-43567-y (PMC6509112; doi:10.1038/s41598-019-43567-y)
Supplement: Supplementary file 2 — Supplementary Material [file 41598_2019_43567_MOESM2_ESM.pdf]

**Integration of bilateral nociceptive inputs tunes spinal and cerebral responses.**

Nabi Rustamov, Stéphane Northon, Jessica Tessier, Hugues Leblond and Mathieu Piché

**Supplementary material**

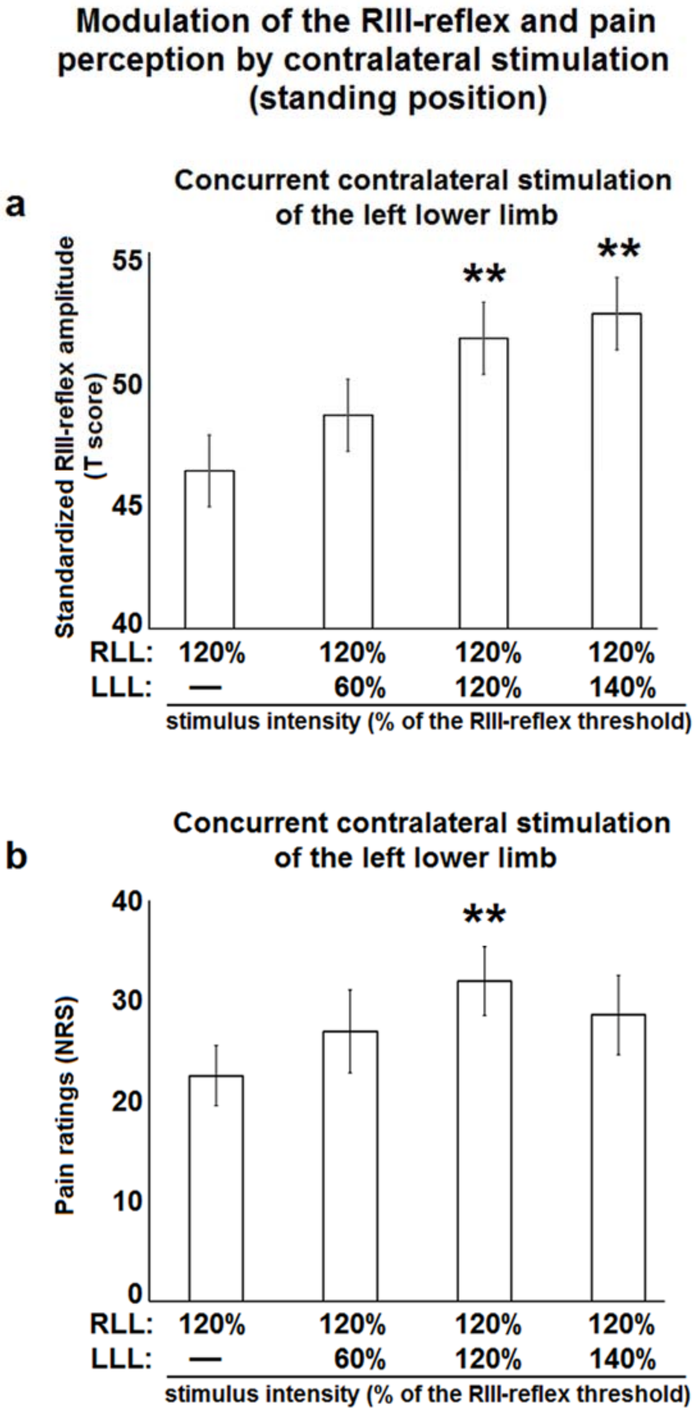


**Supplementary fig. 1 | Modulation of the RIII-reflex amplitude and pain perception by concurrent contralateral stimulation (standing position).**
**a,** The right RIII-reflex was facilitated by concurrent stimulation of the contralateral lower limb: effect of intensity, $F_{3,54} = 6.2$, $p < 0.001$, $\eta_p^2 = 0.27$. Effect of nociceptive (120% and 140% of the left RIII-reflex threshold) contralateral stimulation ($p < 0.004$ and $p < 0.005$, respectively). No effect of non-nociceptive (60% of the left RIII-reflex threshold) contralateral stimulation: $p = 0.09$. RLL, right lower limb; LLL, left lower limb. Error bars, ± 1 SE; x-axis, conditions; y-axis, amplitude (T-score). Significant differences for pairwise comparisons are indicated with symbols (**$p<0.01$ compared with unilateral stimulation). **b,** Right lower limb pain perception was facilitated by concurrent stimulation of the contralateral lower limb: effect of intensity, $F_{3,54} = 3.8$, $p < 0.02$, $\eta_p^2 = 0.18$. Effect of contralateral nociceptive stimulation at 120% of the left RIII-reflex threshold, $p < 0.003$. With contralateral input at 140% of the left RIII-reflex threshold, marginal increase of pain perception: $p = 0.07$. No effect of non-nociceptive contralateral stimulation (60% of the left RIII-reflex threshold): $p = 0.15$. Significant differences for pairwise comparisons are indicated with symbols (**$p<0.01$ compared with unilateral stimulation). RLL, right lower limb; LLL, left lower limb. Error bars, ± 1 SE; x-axis, conditions; y-axis, pain ratings of right sural nerve stimulation (NRS, 0-100).

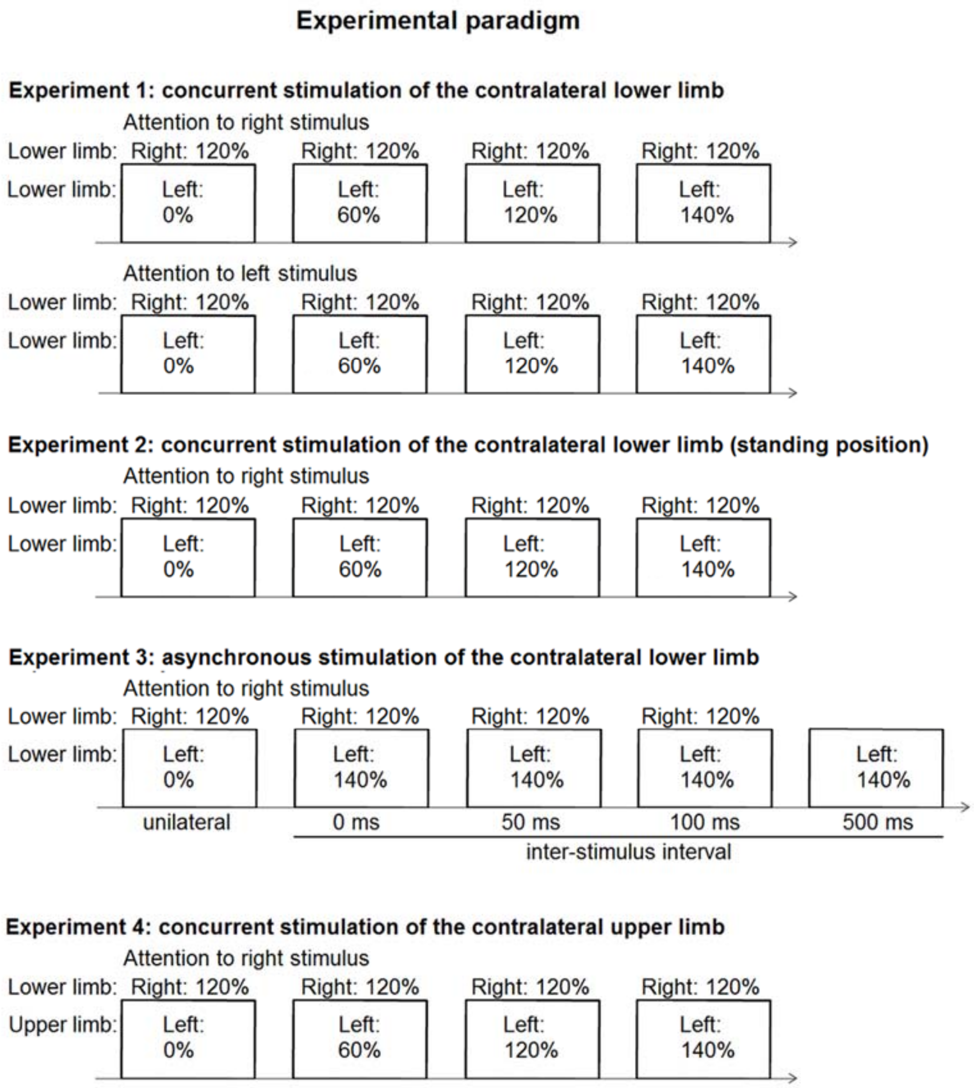


**Supplementary fig. 2 | Experimental paradigm.** Participants underwent four experiments, including concurrent stimulation of lower limbs in a supine (experiment 1) or standing position (experiment 2), asynchronous stimulation of lower limbs (experiment 3) and concurrent stimulation of the right lower limb and contralateral upper limb (experiment 4). In all four experiments, painful electrical stimuli over the right sural nerve was delivered at constant intensity (120% of RIII-reflex threshold). For experiment 1, 2 and 4, contralateral stimulation was applied concurrently at different intensities (60%, 120% or 140% of the left RIII-reflex threshold). For experiment 3, contralateral stimulation was applied at various inter-stimulus intervals of 0 ms, 50 ms, 100 ms or 500 ms with constant intensity of 140% of the left RIII-reflex threshold (experiment 3). In experiment 1, participants were instructed to attend either the right or left stimulus to test the potential effect of selective attention. Participants were prompted to rate shock pain after each stimulation of the right sural nerve. In the experiments, each session included four (experiments 1, 2, 4) or five (experiment 3) blocks of 21 painful electrical stimuli delivered with an inter-stimulus interval of 6-12 seconds varying pseudo-randomly, for a total of 84 to 168 stimuli

depending on the experiment. The order of conditions was counterbalanced to avoid sequence order effects.

**Supplementary vid. 1 | Source estimation of high gamma oscillations represented as t values, based on a voxelwise two-tailed paired t-test on time-frequency source space at 82 Hz.** Time courses of high gamma oscillations induced by bilateral stimulation were compared to those induced by unilateral stimulation (92 ms - 160 ms). The contrast revealed that statistically significant foci of increased high gamma oscillations were located in the lateral prefrontal cortex (maximally over left DLPFC), medial prefrontal cortex, foot region of primary somatosensory cortex (SI), posterior part of ACC extending to the anterior MCC and cingulomotor area. Positive and negative relationships are depicted by warm and cold colors, respectively. Whole-brain t-maps were thresholded at $p<0.05$, false discovery rate corrected for the whole brain.

Table S1 RIII-reflex and pain thresholds (mean ± SEM)

| Experiment | Lower limb | Pain threshold (mA) | RIII-reflex threshold (mA) | Pain and RIII-reflex thresholds correlation |
|---|---|---|---|---|
| 1 | Right | 5.1 ± 0.3 | 7.2 ± 0.4 | r = 0.81; p <0.001 |
| | Left | 5.3 ± 0.3 | 7.1 ± 0.4 | r = 0.79; p <0.001 |
| 2 | Right | 5.1 ± 0.3 | 6.0 ± 0.4 | r = 0.63; p <0.01 |
| | Left | 5.4 ± 0.3 | 6.3 ± 0.4 | r = 0.78; p <0.001 |
| 3 | Right | 5.8 ± 0.3 | 7.5 ± 0.3 | r = 0.66; p <0.01 |
| | Left | 5.8 ± 0.3 | 7.5 ± 0.3 | r = 0.79; p <0.001 |
| 4 | Right | 4.5 ± 0.3 | 5.9 ± 0.4 | r = 0.62; p <0.01 |
